# When nonstandard meets standard: Language and affective dynamics in accent-diverse group interactions

**DOI:** 10.1371/journal.pone.0340873

**Published:** 2026-01-28

**Authors:** Merrisa Lin, Nairán Ramírez-Esparza, Alexandra Paxton

**Affiliations:** 1 Department of Psychological and Brain Sciences, Fairfield University, Fairfield, Connecticut, United States of America; 2 Department of Psychological Sciences, University of Connecticut, Storrs, Connecticut, United States of America; 3 Center for the Ecological Study of Perception and Action, University of Connecticut, Storrs, Connecticut, United States of America; Education University of Hong Kong, HONG KONG

## Abstract

Drawing on communication accommodation theory, this exploratory research examines how the arrival of a standard-accented newcomer influences nonstandard-accented participants’ linguistic patterns and positive affect during group interactions. While prior work often focuses on how newcomers adapt to groups, less is known about how a newcomer shifts existing group dynamics—especially in accent-diverse contexts. Across 49 WebEx group discussions involving 102 nonstandard-accented participants, a standard-accented confederate newcomer joined midway through each session. Study 1 examined changes in participants’ linguistic patterns, including linguistic style matching, speech mistakes, filler words, clout, and authenticity. Results showed that participants generally diverged from each other and converged toward the newcomer in linguistic style. They also made more speech mistakes, exhibited higher clout, and reduced authenticity, although they used fewer filler words—suggesting a disruption to existing interaction dynamics. Study 2 explored whether participants’ positive affect predicted perceived interaction quality after the newcomer’s arrival. Positive affect more strongly predicted perceived interaction quality than negative affect—suggesting that participants view the newcomer as more facilitative than disruptive. These findings highlight a complex dynamic of communication adaptation in accent-diverse interactions, where linguistic shifts are disrupted even as affect remains more positive. Implications for group integration and second-language training are discussed.

## Introduction

Complex group social settings like conferences or community events are often marked by small groups of acquaintances clustering together in conversation. During those events, groups often shift as newcomers join in the conversation. Such interruptions can breathe new energy into the group or disrupt the emerging flow of conversation. Now, imagine the existing group is made up of individuals who speak with a variety of nonstandard accents—what happens when the newcomer has a standard accent?

Past studies have largely focused on how the newcomer assimilates into existing groups [e.g., 1,2] and how groups influence the newcomer [e.g., 3,4]. However, little is known about how existing group members—those who were engaged in the interaction prior to the newcomer’s arrival—might be affected, particularly if they are nonstandard-accented speakers. Given that accents shape social perceptions and power structures in communication [5], understanding how nonstandard-accented speakers respond to the presence of a standard-accented newcomer is critical. In this work, we aim to investigate these under-explored processes within naturalistic interactions.

### Speaker accent, identity, and social perception

As linguistic diversity grows globally, social interactions increasingly involve speakers from varied linguistic and accent backgrounds [e.g., 6,7]. In a given society, accents can be classified based on whether they are considered *standard* or *nonstandard*. *Standard accent* varieties are often used in formal settings and educational systems [8]; they are perceived as lacking distinctive regional, ethnic, or socioeconomic characteristics. *Nonstandard accent* varieties are often linked to specific regions, ethnicities, or nations [9]. Nonnative accent varieties are, by definition, also considered nonstandard. Despite our increasingly global community, nonstandard accents are often perceived less favorably than standard accents [5,10].

Accents often serve as markers of social identity and group affiliations [11]. They support shared meaning-making, shaping social solidarity and community cohesion in diverse societies [7]. Therefore, understanding how nonstandard-accented speakers navigate social interactions is crucial for exploring broader issues related to identity, power, and group membership. Critically, these patterns of communication may differ when nonstandard-accented speakers are engaging with other nonstandard-accented speakers (even if they do not share their specific accent) versus when engaging with standard-accented speakers.

However, accents have been recognized as one of the major causes of miscommunication [12]. This prompts nonstandard-accented speakers to alter the way they speak, including repeating conversations, paraphrasing, or using a different speaking style [13]. Because nonstandard accents are stigmatized [e.g., 14,15], these speakers may adopt a passive role in interactions with standard-accented individuals.

While shared accents may foster solidarity among nonstandard-accented speakers [7], the arrival of a standard-accented newcomer may interrupt this dynamic—not just by introducing a new participant but by altering the social and linguistic hierarchy of the conversation. As cross-accent interactions become more common in professional, educational, and social settings, it is essential to examine how these interactions influence communication dynamics and psychological experiences for nonstandard-accented speakers. Accordingly, this research takes an exploratory approach in understanding how a standard-accented newcomer’s arrival facilitates or disrupts social interactions involving nonstandard-accented speakers, focusing on both participants’ changes in linguistic patterns and positive affect.

### Newcomer arrival and group interaction dynamics

Past research on newcomer and group dynamics has mostly examined how the newcomer assimilates or adjusts to existing groups [e.g., 1,2] and how these groups change the newcomer’s thoughts, feelings, and behaviors [e.g., 3,4]. Relatively less attention has been given to the changes that the newcomer may produce in the group’s structure, dynamics, or performance [16]. Moreover, much of this research has focused on work and organizational settings, primarily measuring team performance and behavioral outcomes [e.g., 1,17]. This leaves a gap in understanding how the newcomer’s arrival changes dynamics in naturalistic social interactions, particularly in terms of their influences on linguistic patterns and positive affect.

Research on newcomer influence suggests that the newcomer can influence the group dynamics [18]. Reviewing across five decades of research in the fields of organizational behavior, management, and psychology, Rink and colleagues [16] documented that the mere arrival of the newcomer can have profound effects on the work behaviors of the existing group, with the newcomer enhancing group creativity [19,20] and fostering innovative outcomes [21]. In these contexts, participants may engage in accommodating behaviors to facilitate the newcomer’s integration, either through closing previous conversations to make way for the newcomer or through summarizing ongoing activities to make sense for the newcomer [22]. However, participants are not necessarily obligated to include the newcomer in social interactions. In fact, participants may be reluctant to immediately accept a newcomer, particularly when the group is cohesive or established. Some exceptions exist, such as when the existing group has a history of failure in their interactions or when the newcomer is socially similar to the participants [16]. These findings highlight the diverse adjustments prompted by the newcomer’s arrival, underscoring its potential to reshape group dynamics in social interactions.

Building on this, prior research has shown that a newcomer’s arrival can influence ongoing interactions in both positive and negative ways [16]. A newcomer may act as a facilitator by bringing fresh perspectives and revitalizing group discussions or disrupting harmony by introducing uncertainty and altering established dynamics [23]. When participants believe that the newcomer will improve group efficiency or help achieve goals, they are more likely to respond positively toward the newcomer [e.g., 24,25]. These positive reactions may facilitate smoother communication between the newcomer and participants, thus improve the quality of interactions. On the other hand, when participants are concerned about interpersonal disruptions and changes in group dynamics following the newcomer’s arrival, they may react less favorably to the newcomer [e.g., 26,27]. This concern can undermine the quality of interactions between the newcomer and the participants. Additionally, research indicated that groups with fluid membership, lower solidarity, and at an early stage of development are more open to the newcomer’s influence, which may lead to more significant changes in group dynamics [18].

Building on these lines of prior work, our study adopts a group context in which nonstandard-accented participants are strangers, grouped by availability rather than shared commonalities, and meeting for the first time. This combination of factors allows us to examine how the arrival of a newcomer influences the interaction dynamics among participants. Specifically, we look to communication accommodation theory [28] to ground our study of a newcomer’s facilitation or disruption in social interactions.

### Communication accommodation theory

Communicative adjustment is a fundamental part of successful social interactions. Communication accommodation theory (CAT) provides a comprehensive framework for understanding how individuals adapt their communicative behaviors both linguistically and affectively [28]. The theory emphasizes both intergroup and interpersonal dynamics in social interactions. It explains how individuals unconsciously adjust their verbal (e.g., accent, speech rate) and nonverbal (e.g., gesture, posture) behaviors to *converge with* (i.e., become more similar to) or *diverge from* (i.e., become more dissimilar to) their conversation partners’ behaviors. Although CAT first emerged in studies of speech and pronunciation, it has since expanded to include studies of all kinds of communicative behaviors, including word choice, grammar, and more [29].

CAT proposes that accommodation generally functions to both index and manage solidarity with a conversational partner, reciprocally and dynamically [30]. These behaviors help facilitate interaction and manage social distance between interactants. Depending on who the interactants are, these changes occur differently [31,32]. CAT identifies two primary types of communication adjustments: convergence and divergence. Convergence is an accommodation strategy, referring to adjusting an individual’s communicative behaviors to be more similar to their interactants’. Divergence is a non-accommodation strategy, referring to adjusting an individual’s communicative behaviors to be more dissimilar to their interactants’ [31]. Moreover, convergence and divergence can each take multiple forms [33]. They are not mutually exclusive and may occur simultaneously depending on various social factors [32].

Diverse motives drive different types of accommodation. For example, people may converge to or reduce variability with their conversational partners to positively reinforce their personal or social identity due to their desire for social approval from their conversational partners [31,34]. It can also be driven by the desire to achieve communicative efficiency [31,34]. Following the similarity-attraction paradigm [35], people are often viewed with social approval if they become communicatively more similar to their interaction partners [36].

By contrast, divergence emphasizes distinctiveness from an individual’s conversation partners as a means to differentiate the speaker from relevant outgroups and assert their own personal or social identity [28]. Divergence may occur particularly when individuals perceive negative attitudes toward their language or speech variety and when positive social identity is violated (e.g., negative bias toward nonstandard accents: [34,37]). This non-accommodation strategy is both an act of differentiation that emphasizes social identity and a process to control interaction.

In interactions involving salient social identities, communication dynamics are shaped by norms, values, stereotypes, behaviors, and the historical affiliations between the identity groups [38]. When interactions occur among strangers, their absence of shared personal history—which often guides interactions among friends and acquaintances—makes salient identity markers (such as accents) more influential in shaping social interactions [39]. Accordingly, in social interactions involving strangers from different accent backgrounds, communication accommodation may emerge as a response to the salient social marker (i.e., accents), rather than pre-existing group cohesion or dynamics.

### Communication accommodation theory and accent

There has been extensive research on language-based social adjustments with communication accommodation theory (for reviews, see [31,40]). However, most studies on accents have focused on standard-accented listener’s perspective, primarily examining how they evaluate and (not) accommodate nonstandard-accented speakers [41]. For instance, Sweeney and Hua [42] investigated how native English speakers adjust their communication in business negotiations with nonnative speakers. Native speakers were instructed to write down what they would say in a negotiation toward an unspecified conversation partner or a nonnative speaker. The authors found that native speakers accommodate nonnative speakers to some extent, such as by simplifying utterances or using more direct phrasing, but these adjustments were inconsistent across speakers. Such findings suggest that standard-accented individuals may accommodate nonstandard-accented speakers, though not universally.

However, fewer studies have examined accommodation among nonstandard-accented speakers. A relevant study explored nonnative speakers’ perceptions of how they are perceived and evaluated by native speakers in Germany [43]. Through semi-structured interviews, the study found that nonnative speakers reported both accommodation (e.g., native speakers providing clarifications when prompted) and non-accommodation (e.g., native speakers oversimplifying language or maintaining rigid speech patterns despite their conversation partner’s cue of incomprehension). While this research offers insights into how nonstandard-accented speakers perceive accommodation from standard-accented individuals, it does not address how nonstandard-accented speakers adjust their communication among themselves or in response to a standard-accented speaker—especially in real-time interactions.

To address this gap, this research applies communication accommodation theory in a novel way to explore how nonstandard-accented speakers adjust both linguistically and affectively when a standard-accented newcomer joins their social interaction. Accommodation can involve observable and measurable changes in speech behavior—such as adopting simpler language structures, mimicking intonation, or matching speech rates [28]—as well as changes in positive affect linked with interpersonal connection and affiliation. These shifts may reflect efforts to connect with others (convergence) or maintain distance (divergence). Importantly, linguistic and affective changes do not always align; speakers may adjust their speech without feeling more engaged, or vice versa [44].

### Communication accommodation theory in the present work

Building on this theoretical groundwork, the present research extends CAT to accent-diverse interactions. Accents function as salient social cues that mark group distinction and guide how individuals manage interpersonal and intergroup interactions [45]. Specifically, how people adjust their communication is a function of their predisposition to construe one another in intergroup terms [32].

In the current studies, we are interested in understanding what happens when a group of nonstandard-accented individuals are suddenly joined by a standard-accented person. To do that, we bring together participants who differ across many characteristics (e.g., race, gender) yet shared the salient identity of speaking with a nonstandard accent in the U.S. This variety reflects the rich range of accents and adds external validity to our work. Although intergroup processes are critical to CAT, accommodation does not require interlocutors to be completely homogeneous or completely heterogeneous; rather, these processes are hypothesized to emerge in relation to the identity that is most salient in the moment. For example, in a landmark study of accommodation processes, Giles and colleagues [46] developed an experiment to emphasize language (French vs. English) as the salient group identity and demonstrated language-specific accommodation processes that did not shift other dynamics (e.g., gender differences, perceived ethnicity). In the present work, we specifically recruit participants who self-identify as having nonstandard accents. This shared experience of navigating nonstandard accents may have guided early interaction patterns before the unexpected arrival of a standard-accented newcomer [e.g., 11]. However, when a standard-accented newcomer enters the interaction, their accent may become a defining cue that reorganizes which identity is most salient for guiding accommodation.

Given the distinctions between convergence and divergence, it is important to consider how nonstandard-accented participants respond to standard-accented newcomer’s arrival in real-time social interactions. CAT posits that speakers adjust their communication depending on whether they appraise the interaction as facilitative or disruptive [40]. Thus, when a newcomer enters with a contrasting accent, group members may shift accommodation targets—from each other to the newcomer. If nonstandard-accented participants perceive the newcomer as facilitating the interaction, they may converge with one another following the standard-accented newcomer’s arrival to gain social approval or liking from the group [31]. In these scenarios, the newcomer’s arrival may facilitate group cohesion [47]. In contrast, if participants perceive the newcomer as disruptive, they may diverge from other participants to differentiate themselves or demonstrate uniqueness in the group. In this case, the newcomer’s arrival may disrupt the existing group interaction [31]. While much of the existing work has examined verbal and nonverbal accommodations in social encounters (for reviews, see [31,40]), few studies have examined how accent dynamics shape linguistic behavior during naturalistic interactions and how interaction partners subsequently reflect on the quality of those interactions.

Therefore, we build on communication accommodation theory in naturalistic group settings. Specifically, we investigate the linguistic patterns of interaction partners in mixed-accent groups (Study 1) and the impact of the conversation on interaction partners’ self-reported affect (Study 2). Together, these studies integrate perspectives on language dynamics and emotional experience, offering a more comprehensive view of how nonstandard-accented speakers navigate social interactions.

## Research and method overview

Our overarching goal is to examine how a standard-accented newcomer’s arrival influences social interactions among nonstandard-accented participants. “Newcomer” refers to the standard-accented speaker who joins an ongoing social interaction among nonstandard-accented individuals. “Participants” are nonstandard-accented individuals who were already engaged in the interaction prior to the newcomer’s arrival. Study 1 explores how linguistic patterns of nonstandard-accented participants change in response to the arrival of a standard-accented newcomer. Study 2 unpacks the positive effect of nonstandard-accented participants. The two studies used different data sources from a single set of naturalistic interactions: Study 1 focused on text-based analysis to examine linguistic changes using interaction transcripts, while Study 2 focused on affect through self-reported survey data. Accordingly, we first introduce the study participants, and the overall design and procedure for naturalistic interactions. Next, we provide separate introductions, study materials, results, and discussions for Study 1 and Study 2. Finally, we conclude with a general discussion.

### Participants

Participants were volunteers recruited from the University of Connecticut (*N* = 102; *M*_age_ = 21.55 years, *SD*_age_ = 5.08; 29.4% men, 68.6% women, 1% non-binary, 1% other). Most participants earned course credits for introductory psychology classes. Some participants earned extra course credits upon agreement with their course instructors. Participants were also recruited through word-of-mouth and advertisements within student organizations. Data were collected between March 20, 2022, and April 20, 2023.

To be eligible, participants needed to be at least 18 years old and to self-identify as nonstandard-accented speakers in the U.S. (Specifically, participants needed to answer “yes” to the following survey question: “Do you have an accent that is not a standard accent commonly heard in North America when speaking English, such as a nonnative accent or regional accent?”). This was partially open to participants’ interpretation. The current study examines how a standard-accented newcomer’s arrival influences the interaction dynamics of nonstandard-accented participants; therefore, participants’ beliefs about their accent’s nonstandardness were key factors in achieving study goals. The question was embedded within a broader demographic questionnaire in a longer screening survey for multiple studies, reducing the likelihood that participants would connect their selection to their accent characteristics.

Participants came from 25 countries across Asia, Africa, North America, Central and South America, the Caribbean, and Europe. Taken together, they reported 29 unique native languages on a multi-select multiple choice question. The most common native language was Chinese (including Mandarin, Cantonese, Taiwanese, etc.), spoken by 27 participants (26.5%). Participants lived in the U.S. for an average of 6.05 years (*SD* = 6.55). All participants were undergraduates, except for 20 (19.6%) who indicated that they were pursuing postgraduate degrees. Regarding participants’ racial backgrounds, they responded to a multi-select multiple-choice question. The majority identified as Asian (*n* = 52; 51.0%), followed by White (*n* = 27; 26.5%), Black or African American (*n* = 18; 17.6%), and more than one race (*n* = 5; 4.9%). Most participants identified as non-Hispanic or non-Latine (*n* = 87; 85.3%). When asked about their socioeconomic status on a 5-point Likert scale ranging from 1 (*working class*) to 5 (*upper class*), participants self-reported an average of 2.60 (*SD* = 1.14). See Table 1 for a summary of participants’ sociodemographic characteristics.

**Table 1:** Sociodemographic characteristics of participants.

Characteristics	*n*	%
Gender		
Man	30	29.4%
Woman	70	68.6%
Non-Binary	1	1.0%
Others	1	1.0%
Race		
White	27	26.5%
Asian	52	51.0%
Black or African American	18	17.6%
More than One Race	5	4.9%
Ethnicity		
Hispanic or Latine	87	85.3%
Non-Hispanic or Non-Latine	15	14.7%
Education Level		
College Freshman	36	35.3%
College Sophomore	21	20.6%
College Junior	20	19.6%
College Senior	5	4.9%
Graduate Degree	20	19.6%
Home Country		
Bangladesh	2	2.0%
Brazil	2	2.0%
China	25	25.0%
Germany	2	2.0%
Ghana	3	3.0%
India	9	9.0%
Nigeria	5	5.0%
South Korea	3	3.0%
Taiwan	2	2.0%
USA	31	31.0%
Vietnam	2	2.0%
Other Countries^a^	14	14.0%
Native Language		
English	23	22.5%
Chinese	27	26.5%
Spanish	11	10.8%
Korean	4	3.9%
German	2	2.0%
Portuguese	2	2.0%
Yoruba	2	2.0%
Gujrati	2	2.0%
Vietnamese	3	2.9%
Bengali	5	4.9%
Hindi	3	2.9%
Other Languages^b^	18	17.6%

Participants were on average 21.55 years old (*SD* = 5.08) and had been living in the U.S. for an average of 6.05 years (*SD* = 6.55). Their socioeconomic status was 2.60 (*SD* = 1.14) on a 5-point scale, higher scores indicate higher socioeconomic status.

^a^For 14 home countries, only one participant was included. To reduce identifiability, these countries are not reported separately.

^b^For 18 native languages, only one participant reported speaking each language. To reduce identifiability, these languages are not reported separately.

### Design and procedure

We developed an innovative naturalistic method in which nonstandard-accented strangers were asked to discuss university student life in small groups over WebEx, an online conferencing platform designed to facilitate real-time communication among a group of individuals, allowing participants to connect and interact regardless of their physical locations. The platform also has an embedded recording function in which participants’ interactions can be recorded without interfering with the discussions [48]. WebEx was also the primary online conferencing software for the University of Connecticut. Thus, we selected this software to improve the likelihood that our participants would be highly familiar with the platform. After prescreening, eligible participants read an online consent form and provided informed consent via an electronic signature. Then, they completed a demographic questionnaire. The questions include gender, race, ethnicity, language backgrounds, length of residence in the U.S., education, and home country. Then, we matched participants’ schedules to arrange real-time WebEx social interactions. Due to specific eligibility criteria and participant availability constraints, we anticipated difficulty in recruitment. To ensure adequate group sizes, two undergraduate students repeatedly participated in the WebEx sessions as mock participants. To confirm that their presence did not systematically influence group behavior, we conducted additional analyses including the mock participants. Results remained largely consistent (see S1 File Supporting Information for details on Recruitment Adjustments). In total, 49 WebEx discussion sessions were conducted.

Prior to each WebEx session, we instructed participants to join the video call from a quiet area where they could engage in conversation without distractions. We also asked them to ensure that their audio and camera were functional. During the interaction, the experimenter instructed the participants to discuss their university lives with one another for 20 minutes. The experimenter stated: “In the next 20 minutes, please chat with each other about your lives at the university. Here are some examples you can chat about: the organizations you are involved in, the courses you are taking, job experiences, and many more.”

Halfway through the discussion, a standard-accented newcomer joined the discussion (i.e., at the 10-minute mark), pretending to be a late participant. This newcomer, who was a confederate, spoke with a standard American English accent (see S1 File Supporting Information for details on Newcomer Backgrounds). Upon joining, the newcomer was scripted to say, “Sorry I am late; my class went overtime!” Thereafter, the newcomer was instructed to follow the ongoing flow of discussion and integrate naturally. In instances where the newcomer initiated a new topic, they were directed to focus on school life specific to classes and extracurricular activities. With this design, we created two phases of interaction: the first was pre-newcomer’s arrival, involving only nonstandard-accented participants, and the second was post-newcomer’s arrival, introducing a standard-accented newcomer.

**Fig 1:**
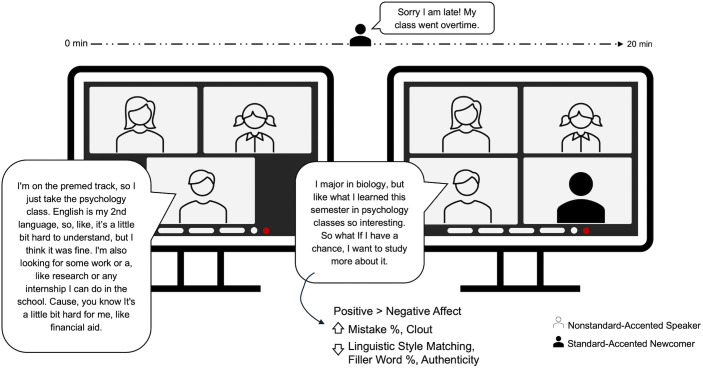
Study design and procedure overview.

After the discussion, participants completed a follow-up questionnaire about the interaction they just had. The questions included perceived quality of interaction [49], and positive and negative affect toward the interaction [50]. Upon completion, participants were debriefed. See Fig 1 for study design and procedure overview.

### Ethics statement

This study received approval from the University of Connecticut Institutional Review Board (IRB). All procedures were conducted in accordance with the IRB’s ethical guidelines. Participants’ written informed consent was obtained electronically from all participants prior to study sessions. Eligible participants reviewed an online consent form and provided their consent via an electronic signature after prescreening.

## Study 1: Linguistic patterns

We first explore whether the arrival of a standard-accented newcomer facilitates or disrupts the social interaction dynamics among nonstandard-accented participants, as reflected in linguistic patterns. Specifically, we examine changes in participants’ use of function words, mistakes, filler words, speech clout, and authenticity.

### Linguistic style matching (LSM)

Past studies have demonstrated that communication accommodation can be displayed in shifts in linguistic patterns. One such pattern is the convergence of function words within an interaction. *Linguistic style matching* measures the extent to which an individual’s function word usage covaries with that of others [51]. These measures serve as sensitive indicators of both conversational engagement and social dynamics, such as dominance and rapport, that go beyond self-reports. Consistent with the communication accommodation theory, linguistic style matching can assess the degree to which individuals adapt to others in novel social interactions [51], reflecting interpersonal similarity of psychological states [52–54].

Linguistic style matching primarily focuses on the use of function words. Function words (e.g., pronouns, articles) are typically short, frequently used, and have little meaning outside the context of a sentence [52,55]. As a result, these words are processed rapidly, largely unconsciously, and require shared social knowledge, or common ground, to be used effectively [56,57]. This unobtrusive measure of nonconscious verbal matching allows us to assess interpersonal language similarity irrespective of context.

Since our analyses are exploratory, we did not have specific hypotheses. Instead, we anticipated that we could see one of two alternative patterns—that LSM could either increase or decrease, reflecting different accommodation processes. An increase in LSM among participants after the standard-accented newcomer joins would be consistent with convergence-related behavior, suggesting that the newcomer may facilitate the interaction. In contrast, a decrease in LSM between participants after the newcomer arrives would be consistent with divergence-related behavior, suggesting that the newcomer may disrupt the interaction. To examine these dynamics, we analyze linguistic style matching at two levels: (1) between a participant and all other participants in the same WebEx session (groupwise analysis) and (2) between each pair of participants in the same session (pairwise analysis). In all analyses, we remove any turns from confederates (i.e., the newcomer and mock participants), so that our analyses only reflect the language spoken by actual participants.

### Speech disfluencies

We also examine the changes in speech disfluencies (i.e., mistakes and filler words). While speech disfluencies are not direct components of communication accommodation, they may serve as linguistic markers of social integration (facilitation) or distancing (disruption). Speech disfluencies can provide insights into whether participants use convergence or divergence strategies in interaction [e.g., 58]. McGlone and Pfiester [58] found that when individuals are reminded of their self-relevant gender stereotypes, which contribute to mental disturbance and heightened anxiety, their speech is marked with more disfluencies (e.g., more errors, more filler words, more interruptions).

Building on this preliminary evidence, we approached the present analyses with the following expectations. If the standard-accented newcomer’s arrival disrupts the conversation among nonstandard-accented participants (e.g., if the standard accent is more salient), participants may experience uncertainty and diminished fluency, leading to more speech mistakes and increased filler words (e.g., oh, um). On the other hand, if the newcomer is seen as facilitating interaction, participants may use the same amount or even fewer speech mistakes or reduced filler words, reflecting similar or increased ease.

### Communication dynamics metrics

Beyond speech disfluencies, communication dynamics metrics such as clout and authenticity may provide additional insight into how the newcomer’s arrival influences participants’ linguistic patterns. Although clout and authenticity are not direct indicators of communication accommodation, they share similar underlying social and psychological adaptation processes. As such, we aim to use the individual language patterns during these conversations to get a fuller glimpse of nonstandard-accented speakers’ experiences.

*Clout* entails the exhibited levels of confidence, social status, and leadership in speech [59,60]. High-status individuals (e.g., military leaders, professors, and group leaders) tend to use more “we” and “you”, while lower-status individuals rely more on “I” statements [61]. This may be because, during group interactions, status is associated with attentional biases—higher-ranking individuals tend to focus on others to facilitate collective decision-making, while lower-ranking individuals are more self-focused, concerned with managing impressions and seeking to gain the leader’s approval [61]. For instance, Olympians who more frequently use “you” and “we” on social media posts (e.g., “We are proud to represent Team USA”) are perceived as higher in clout, whereas those who use more first-person and impersonal singular pronouns (e.g., “It is a dream for one to represent Team USA”) are rated as lower clout [62 pp. 297–298]. This evidence suggests that language conveying clout can signal authority, control, and social dominance in a conversation.

Accordingly, our expectations for clout are as follows: If participants perceive the newcomer as facilitating interaction, they may seek social approval and avoid asserting dominance. Thus, they may use less clout-related language that fosters social cohesion—for instance, relying more on self-focused references (e.g., “I”) rather than collective pronouns to appear deferential and agreeable. In contrast, if the newcomer’s presence is disruptive, participants may attempt to reassert their status and dominance by using more clout-related words. They may focus on the collective pronouns (e.g., “we”, “you”) that emphasize authority and influence within the group.

*Authenticity* reflects how open and spontaneous a person’s speech is. High authenticity is associated with spontaneous, genuine, and personal speech, while low authenticity suggests guarded and socially cautious communication [59,60]. High authenticity is indexed by self-references (e.g., “I”, “me”, “mine”), insight words (e.g., “aware”, “think”, “know”), differentiation words (e.g., “but”, “except”, “without”), and relativity terms (e.g., “above”, “now”, “near”). Low-authenticity indices include discrepancies from reality (e.g., “must”, “should”, “could”) and third-person singular pronouns (e.g., “she”, “her”, “hers”) [63–65]. For example, in Olympians’ social media posts, “I am here to bring home the gold medal” signals a high authenticity through using “I” and relativity word “here.” In contrast, “It would have been ideal for my team to bring home the gold medal” signals low authenticity due to the usage of “would have been,” indexing discrepancies from reality [62 p. 297]. Importantly, lower authenticity does not imply dishonesty—it may instead signal self-monitoring, uncertainty, and efforts to manage impressions.

Accordingly, we propose the following expectations for authenticity. If the newcomer’s arrival is perceived as facilitative, participants may feel more comfortable expressing their thoughts, leading to increased authenticity in speech—a potential marker of social cohesion. On the other hand, if the newcomer’s arrival is perceived as disruptive, participants may be more self-conscious about their speech, leading to decreased authenticity, suggesting heightened self-monitoring and social distancing.

### Study materials

#### Transcript of WebEx social interaction.

To quantify participants’ language dynamics effectively and accessibly, we recorded their real-time social interactions and used WebEx’s built-in AI transcription service to automatically transcribe the sessions. AI-based transcription tools are increasingly adopted in qualitative and communication research due to their efficiency and growing accuracy, which in many cases approach that of human transcription [66,67]. The software employs speech recognition algorithms and natural language processing that allow us to detect subtle differences in speech patterns. To analyze linguistic changes, we split the transcripts into two segments: before and after the newcomer’s arrival. We acknowledge that automated transcription may introduce errors or reflect algorithmic biases, thus, we implemented a hybrid approach combining AI and human validation. All transcripts were reviewed by trained research assistants. For variables like linguistic style matching, clout, and authenticity, transcript errors were corrected to ensure accurate analysis. For features where the presence of disfluency was central (i.e., speech mistakes and filler words), we preserved the original utterances and annotated them manually (as described below). This combined approach balances ecological validity with analytic rigor. Additional details about the analytic procedures and the coding of transcripts are provided in the following section.

#### Analytic procedures and coding.

We used the features and internal dictionaries of the Linguistic Inquiry and Word Count (LIWC [59,60]) to perform text-based analysis on the transcripts of WebEx social interactions. LIWC is a dictionary-based text analytic program designed to count the occurrences of words within specified categories in text files [59,60]. This approach is based on the understanding that words people use convey psychological information beyond their literal meaning. By counting the words people use that correspond to grammatically or psychologically meaningful linguistic dimensions (e.g., function words, emotion), we can gauge the extent to which individuals’ orientations reflect these dimensions [68,69]. LIWC is a well-established text analysis program designed to conduct such analyses. In addition to LIWC’s internal dictionaries, the program also allows user-created dictionaries, which guide LIWC in searching for words of interest (e.g., mistakes, filler words).

Importantly, although LIWC has been most widely applied to written text, past research has also extended its use to real-time dyad and group conversations [e.g., 70]. We acknowledge that spoken and written language differ in structure, and that LIWC’s dictionaries may not always capture subjective experiences in everyday speech [e.g., 71]. However, the latest version of LIWC [59] used in the present work addresses this limitation by expanding its corpus to include transcribed natural conversations primarily composed of face-to-face social interactions between two or more people who may or may not know each other [e.g., 51,61]. This update enhances LIWC’s ecological validity and makes it conceptually well aligned with our study’s focus on real-time conversational dynamics.

To examine the changes in linguistic patterns, we coded transcripts for three primary categories: (1) linguistic style matching; (2) proportion of mistakes and filler words; and (3) speech clout and authenticity.

***Linguistic Style Matching****:* First, to examine how participants’ linguistic styles matched with those of other participants in their WebEx session, we analyzed function words using LIWC. Examples of function words include articles (e.g., “an,” “the”), prepositions (e.g., “of,” “for”), conjunctions (e.g., “and,” “but”), adverbs (e.g., “about,” “just”), and personal pronouns (e.g., “I,” “you”). According to Niederhoffer and Pennebaker [51], linguistic style matching can be explored in two general ways. The first is to examine whether people’s languages are synchronous on a turn-by-turn level, while the second is to conduct between-subjects analyses, comparing an individual’s language use to that of their interaction partners. Given our focus on the influence of the newcomer’s arrival on participants, we adopted the second approach at both the groupwise and pairwise levels. At the groupwise level, we compared the degree of linguistic style matching between a participant and all other participants in the same WebEx session (i.e., one-to-many). At the pairwise level, we compared the linguistic style matching between a participant and another participant in the same WebEx session (i.e., one-to-one).

To calculate LSM, we first segmented transcripts by speaker, creating a dataset where each row represents a participant, with separate columns for speech before and after the newcomer’s arrival. Then, we analyzed the texts with LIWC. LIWC computed the percentage of words in a text that fall into eight basic-level function word categories (see Table 2). For each function word category, we calculated LSM scores using the standard formula. Equation (1) shows an example of calculating LSM of prepositions:

**Table 2:** Word categories for measuring linguistic style matching.

Category	Examples
Personal Pronouns	I, you, my, me
Impersonal Pronouns	that, it, this, what
Articles	a, an, the
Conjunctions	and, but, so, as
Prepositions	to, of, in, for
Auxiliary Verbs	is, was, be, have
Adverbs	so, just, about, there
Negations	not, no, never, nothing

The measure of linguistic style matching and word categories are based on the 2022 version of Linguistic Inquiry and Word Count (LIWC [59,60]).


$${LSM}_{\text{preps}}=\text{1}–[(|{preps}_{\text{1}}–{preps}_{\text{2}}|)/({preps}_{\text{1}}+{preps}_{\text{2}}+\text{0}.\text{0}\text{0}\text{0}\text{1})]$$ (1)

Here, *preps*_1_ represents the percentage of prepositions (relative to all words spoken) used by a given participant, and *preps*_2_ is either (1) the average percentage used by all other participants in the WebEx session (groupwise analysis; one-to-many) or (2) the percentage used by the paired participant (pairwise analysis; one-to-one). Accordingly, we calculated two types of LSM scores—groupwise and pairwise. The denominator includes 0.0001 to prevent division by zero. For both groupwise and pairwise analyses, the eight category-level LSM scores were averaged to produce a composite LSM score ranging from 0 to 1, with higher scores indicating greater linguistic similarity between a participant and a target partner [52,53]. We tested both types of LSM scores across three analysis models; model specifications are detailed in the Results section.

***Speech Disfluencies***: Second, mistakes and filler words were coded by two native English-speaking coders who were blind to the research design and goals. They were instructed to watch the WebEx recordings and coded for two separate linguistic qualities. To identify speech mistakes, coders cross-referenced the transcripts for contents that were not correctly transcribed. This included incorrect spellings, misidentified words, and missing words, which the coders marked as mistakes (e.g., if WebEx transcribed an utterance as “insight” instead of “psych”). For filler words, the coders identified words, phrases, or sounds that convey little to no meaning but mark a pause or hesitation in speech, such as *um*, *uh*, *er*, *ah*, *okay*, and *yeah*. These filler words signal speech disfluency, suggesting the speaker needs a moment to gather their thoughts and may be unsure about what they are saying [72].

Upon comparing the coding results from Coder 1 and Coder 2, we identified significant discrepancies in Coder 1’s interpretation of the coding scheme. As a result, we relied on Coder 2’s coding for further analysis, with the first author reviewing all coded transcripts for consistency. Minor discrepancies were discussed and resolved through a review process with all coauthors, ensuring that the final coding was consistent and accurate. In the transcripts, mistakes were labeled with “EE” and filler words with “FF” (e.g., “EEinsight”). Then, we created dictionaries in LIWC to identify these labels, enabling the program to tally the number of mistakes and filler words participants used before and after the newcomer’s arrival. Further details on the coding scheme and intercoder reliability can be found in S1 File Supporting Information.

***Clout and Authenticity***: Third, participants’ speech clout and authenticity before and after the newcomer’s arrival were assessed using LIWC’s internal summary measures. These measures are calculated using LIWC’s proprietary algorithms and reported as standardized percentile scores ranging from 1 to 99 [59,60]. Although the specifics of how these measures are calculated are not disclosed from LIWC, Table 3 provides example words associated with clout and authenticity.

**Table 3:** Example words of LIWC clout and authenticity composite measures.

Example Word Category	Example Words	Association
Clout		
First-Person Singular Pronouns	I, me, my, myself	–
First-Person Plural Pronouns	we, our, us, lets	+
Second-Person Pronouns	you, your, yourself, u	+
Authenticity		
First-Person Pronouns	I, me, my, mine	+
Insight	aware, think, know, feel	+
Differentiation	but, except, without, if	+
Relative	above, now, near, here	+
Discrepancy	must, should, could, would	–
Third-Person Pronouns	she, he, her, his	–

In the Association column, “+” indicates positive association with clout or authenticity and “-” indicates a negative association. The clout and authenticity composite measures are computed using the 2022 version of Linguistic Inquiry and Word Count (LIWC [59,60]).

### Results

To test whether the newcomer facilitated or disrupted the social interaction through linguistic patterns, we performed a series of generalized linear mixed models (GLMM) and linear mixed models (LMM), with linguistic style matching, the percentage of mistakes, the percentage of filler words, speech clout, and speech authenticity as the dependent variables, respectively. Mixed-effects models were used to account for both fixed effects (e.g., the newcomer’s arrival) and random effects (e.g., individual participant or WebEx session variability). This approach is well-suited for the non-independence of observations, such as repeated measures on the same participants and allows for capturing session-level variability, making it suitable for analyzing complex data structures like in the current study. Below, we present our results, organized according to the three linguistic categories. For clarity and readability, we include full results for most models in tables rather than in the text.

#### Investigating confounds.

To test for the potential confounding effects of individual differences among standard-accented newcomers, we conducted post-hoc linguistic style matching analyses of the newcomers with their groups. All newcomers showed acceptable similarities in their linguistic styles (see S1 File Supporting Information for details). This suggests that any observed effects are unlikely to be driven by systematic differences in the standard-accented newcomer’s behavior across groups.

#### Linguistic style matching.

We performed two sets of analyses, groupwise and pairwise linguistic style matching. In each set of analysis, we conducted three analysis models to test the changes in participants’ linguistic style matching (with all other participants or with the paired participant in the same WebEx session) and examine the influence of the newcomer.

***Analysis Model 1: Changes of Participants’ LSM:*** In the first analysis model, we performed GLMMs to examine whether participants’ linguistic style matching changed after a standard-accented newcomer entered the conversation. Given that LSM is a proportionate measure bounded between 0 and 1, GLMM analysis with a beta distribution and a logit link function was appropriate for the analysis model. We performed the analyses in R [73] using the *glmmTMB* package [74].

In the groupwise analysis, we specified the conversation segment relative to the newcomer’s arrival (i.e., before vs. after) as the fixed effect, and we included random intercepts to account for individual participant variability. LSM was significantly and negatively predicted by the newcomer’s presence. Participants’ language use became much less similar to other nonstandard-accented participants after the newcomer’s arrival (before: *M* = 0.84, *SD* = 0.15; after: *M* = 0.79, *SD* = 0.17), suggesting a divergence in linguistic patterns. The full analysis model summary is available in Table 4 A-1.

**Table 4:** (A-1) Groupwise analysis of linguistic style matching: Analysis Model 1. (A-2) Pairwise analysis of linguistic style matching: Analysis Model 1.

**A-1. Groupwise analysis of linguistic style matching: Analysis Model 1**						
*Predictors*	*Estimates*	*SE*	*β*	*95% CI* *[LL, UL]*	*z*	*p*
Linguistic Style Matching (LSM) Among Participants						
(Intercept)	2.11	0.17	1.52	[1.76, 2.45]	12.10	<.001
Newcomer’s Arrival^a^	−0.39	0.10	−0.20	[-0.58, -0.20]	−4.01	<.001
**Random Effects**						
Individual Participant Variance	0.32					
Marginal R^2^ / Conditional R^2^	0.101/ 0.959					
**A-2. Pairwise analysis of linguistic style matching: Analysis Model 1**						
*Predictors*	*Estimates*	*SE*	*β*	*95% CI* *[LL, UL]*	*z*	*p*
Linguistic Style Matching (LSM) of Each Participant Pair						
(Intercept)	1.10	0.32	0.74	[0.48, 1.72]	3.47	<.001
Newcomer’s Arrival^a^	−0.24	0.18	−0.12	[-0.60, 0.95]	−1.31	.192
**Random Effects**						
WebEx Session Variance	0.35					
Marginal R^2^ / Conditional R^2^	0.030/ 0.754					

*SE* = standard error, *β* = standardized coefficient, CI = confidence interval, *LL* = lower limit, *UL* = upper limit, *z* = z-score, *p* = p-value.

^a^1 = before newcomer’s arrival, 2 = after the newcomer’s arrival.

In the pairwise analysis, the fixed effect was the conversation segment relative to the newcomer’s arrival (i.e., before vs. after), and the random effect was the WebEx session. The results indicated that newcomer’s arrival was not a significant predictor of linguistic style matching at the pairwise level. However, although it did not reach statistical significance, the direction of the change was consistent with the patterns in the group-level analyses (before: *M* = 0.78, *SD* = 0.19; after: *M* = 0.70, *SD* = 0.24). The full analysis model summary is available in Table 4 A-2.

**Fig 2:**
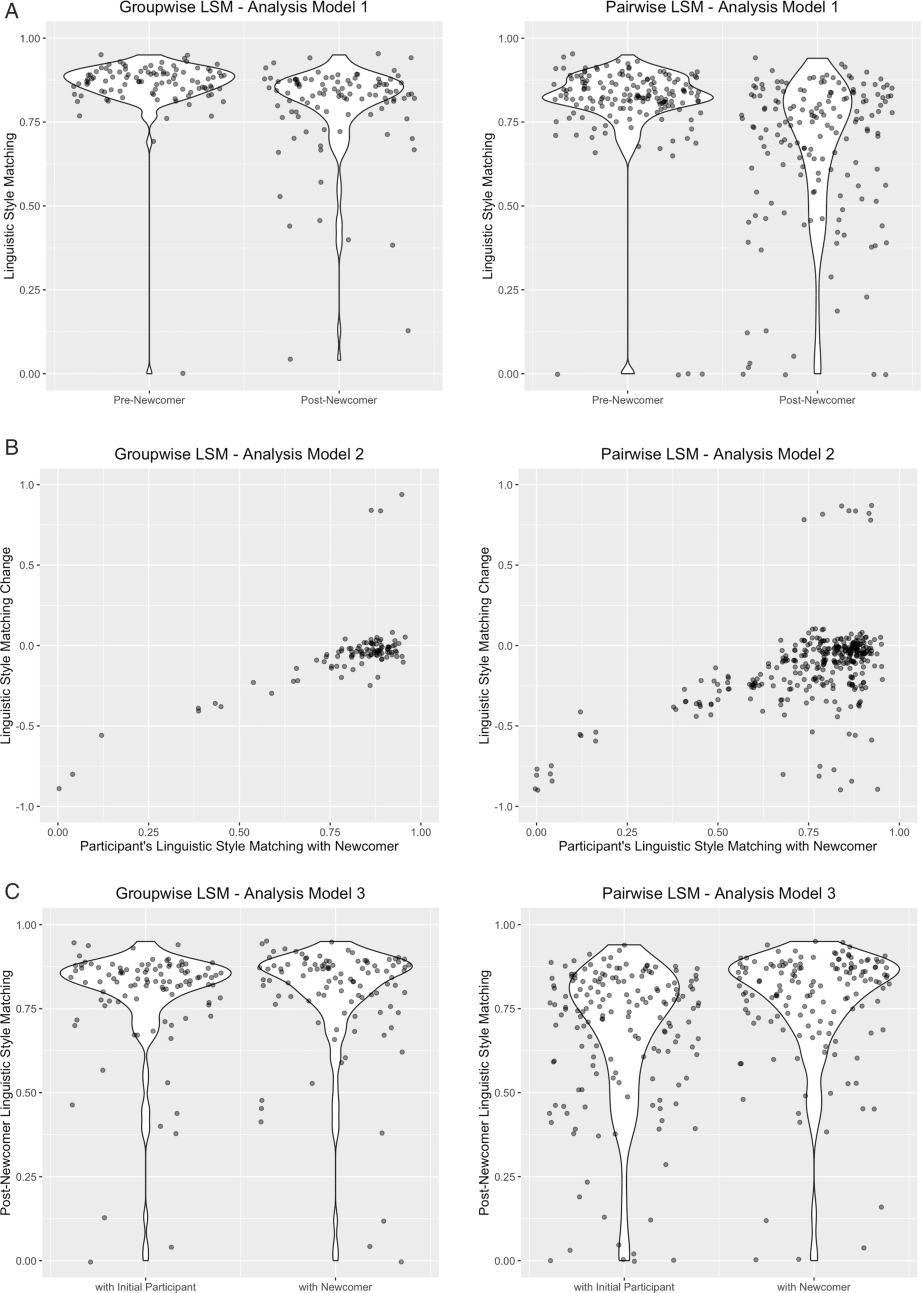
(A) Analysis Model 1—Changes of participants’ LSM. (B) Analysis Model 2—The newcomer’s influences on changes of participants’ LSM. (C) Analysis Model 3—Impact of newcomer’s presence on participants’ LSM Post-arrival.

Although the pairwise analysis results were not statistically significant, both groupwise and pairwise analyses showed a trend toward decreased linguistic style matching, suggesting linguistic divergence. After the newcomer joined, participants significantly diverged in language style from other nonstandard-accented participants in the same WebEx session at the group level. Importantly, this is only accounting for participants’ linguistic interactions with one another; it does not include participants’ interactions with the newcomer or mock participants. Each participant pair also showed a directional trend toward divergence at the pairwise level, although it did not reach statistical significance. The stronger effect observed in the groupwise analysis may be due to the averaging of LSM scores, leading to reduced variability. In contrast, the greater individual variability in the pairwise analysis may have diluted the effect of newcomer’s arrival. Overall, these findings suggest that the newcomer’s arrival disrupted the existing social interaction, leading to greater linguistic divergence among participants (see Fig 2A for results visualization).

***Analysis Model 2: The Newcomer’s Influences on Changes of Participants’ LSM:*** In the second analysis model, we tested whether the newcomer’s linguistic style contributed to changes in participants’ linguistic style matching. We performed two separate LMMs using the *lme4* package in R [75]. The dependent variable was the change of participants’ LSM with other participants in the same session (groupwise analysis) or with each possible pair of participants in the same session (pairwise analysis). The predictor was the LSM between the target participant and the newcomer. We also included a random intercept to account for WebEx session variability in each analysis model.

Groupwise analysis results showed that the changes in LSM among participants was significantly predicted by each participant’s LSM with the newcomer. The more participants matched the newcomer’s linguistic style, the greater their divergence from the group of other participants. The full analysis model summary is available in Table 5 B-1. Similarly, the pairwise analysis results showed that the changes in LSM between each pair of participants was significantly predicted by their LSM with the newcomer. That is, the more participants matched the newcomer’s linguistic style, the greater their divergence from their paired participant. The full analysis model summary is available in Table 5 B-2.

**Table 5:** (B-1) Groupwise analysis of linguistic style matching: Analysis Model 2. (B-2) Pairwise analysis of linguistic style matching: Analysis Model 2.

**B-1. Groupwise analysis of linguistic style matching: Analysis Model 2.**						
*Predictors*	*Estimates*	*SE*	*β*	*95% CI* *[LL, UL]*	*t*	*p*
Change of Linguistic Style Matching (LSM) Among Participants						
(Intercept)	−0.75	0.07	0.09	[-0.88, -0.61]	−11.20	<.001
Participant and Newcomer LSM	0.85	0.08	0.06	[0.69, 1.00]	10.90	<.001
**Random Effects**						
WebEx Session Variance	0.01					
Marginal R^2^ / Conditional R^2^	0.453/ 0.725					
**B-2. Pairwise analysis of linguistic style matching: Analysis Model 2.**						
Change of Linguistic Style Matching (LSM) of Each Participant Pair						
(Intercept)	−0.44	0.08	0.04	[-0.60, -0.28]	−5.35	<.001
Participant and Newcomer LSM	0.45	0.09	0.30	[0.27, 0.64]	4.82	<.001
**Random Effects**						
WebEx Session Variances	0.04					
Marginal R^2^ / Conditional R^2^	0.105/ 0.585					

*SE* = standard error, *β* = standardized coefficient, CI = confidence interval, *LL* = lower limit, *UL* = upper limit, *t* = t-value, *p* = p-value.

Both groupwise and pairwise analyses revealed a consistent pattern. LSM with the newcomer significantly predicted changes in participant’s LSM, both with other participants in the same session (groupwise analysis) and within individual participant pairs in the session (pairwise analysis). Participants who adapted more to the newcomer’s linguistic style simultaneously diverged from other participants (see Fig 2B for results visualization).

***Analysis Model 3: Impact of Newcomer’s Presence on Participants’ LSM Post-Arrival:*** In the third analysis model, we conducted two separate GLMMs to further unpack how the newcomer influenced the linguistic style of participants after their arrival. The analyses were conducted using the *glmmTMB* package in R [74]. We calculated LSM among participants and the newcomer after the newcomer’s arrival, and we predicted these values with partner type as the fixed effect (i.e., with other participants versus with the newcomer). Again, we created one groupwise model (to account for each participant’s average LSM with the rest of the participants in the WebEx session versus with the newcomer) and one pairwise model (to account for each participant’s individual LSM with each other person in the WebEx session versus with the newcomer). Again, random intercepts were included to account for WebEx session variability.

Groupwise analysis results showed that the type of LSM did not significantly predict post-newcomer LSM. The full analysis model summary is available in Table 6 C-1.

**Table 6:** (C-1) Groupwise analysis of linguistic style matching: Analysis Model 3. (C-2) Pairwise analysis of linguistic style matching: Analysis Model 3.

**C-1. Groupwise analysis of linguistic style matching: Analysis Model 3**						
*Predictors*	*Estimates*	*SE*	*β*	*95% CI* *[LL, UL]*	*z*	*p*
Post-Newcomer Arrival Linguistic Style Matching (LSM)						
(Intercept)	1.18	0.12	1.19	[0.94, 1.41]	9.77	<.001
LSM Type^a^	0.04	0.11	0.02	[-0.18, 0.27]	0.39	.699
**Random Effects**						
WebEx Session Variance	0.34					
Marginal R^2^ / Conditional R^2^	0.001/ 0.896					
**C-2. Pairwise analysis of linguistic style matching: Analysis Model 3**						
Post-Newcomer Arrival Linguistic Style Matching (LSM) of Each Participant Pair						
(Intercept)	0.87	0.15	1.10	[0.63, 0.37]	5.86	<.001
LSM Type^b^	0.37	0.13	0.18	[0.12, 0.63]	2.88	<.010
**Random Effects**						
WebEx Session Variances	0.48					
Marginal R^2^ / Conditional R^2^	0.060/ 0.918					

*SE* = standard error, *β* = standardized coefficient, CI = confidence interval, *LL* = lower limit, *UL* = upper limit, *z* = z-score, *p* = p-value.

^a^1 = LSM among participants, 2 = LSM between participant and the newcomer.

^b^0 = LSM between paired participants, 1 = LSM between participant and the newcomer.

In the pairwise analysis, post-newcomer LSM significantly varied by partner type. In other words, after the newcomer arrived, participants showed significantly greater convergence with the newcomer than with other participants. The full analysis model summary is available in Table 6 C-2.

Although the groupwise analysis was not significant, both groupwise and pairwise analyses showed similar trends. The pairwise analysis showed that participants converged more with the newcomer than with other participants, but we did not find a significant effect in the groupwise analysis. This suggests that individual variability is greater in the pairwise model, allowing for a more pronounced effect of convergence toward the newcomer. By contrast, it may be that the comparison of group-level linguistic patterns could have washed out pairwise variability, as their patterns already converged with the newcomer. Taken together, this pattern suggests that the newcomer disrupted the interaction and drove participants to converge to their linguistic styles (see Fig 2C for results visualization).

#### Speech disfluencies.

Next, to complement the findings on LSM, we conducted a series of GLMMs and LMMs to analyze the differences in participants’ use of mistakes and filler words before and after the newcomer’s arrival. Since the newcomer introduces an additional interaction partner, participants may naturally reduce the number of words they say due to limited speaking turns. Thus, the usage of mistakes and filler words was calculated as percentages of words spoken during each segment and transformed to a 0–1 range.

For the percentage of mistakes and filler words, we performed two analysis models, respectively. First, we conducted a GLMM with a beta distribution and a logit link function to test the differences before and after the newcomer’s arrival, and we included participant variability as the random effect. For the second analysis model, we conducted a LMM to examine whether the newcomer’s target linguistic behavior predicted changes in the participants’ target behavior.

The percentage of mistakes was significantly predicted by the newcomer’s arrival (see Table 7A). Participants made significantly more mistakes after the newcomer’s arrival (before: *M* = 13.46, *SD* = 7.25; after: *M* = 16.89, *SD* = 12.87). However, participants’ percentages of mistakes were not significantly predicted by the newcomer’s mistake percentage (see Table 7B). Taken together, our results suggest that the newcomer’s presence caused general disruption: Participants made more mistakes after the newcomer arrived, regardless of the newcomer’s patterns of mistakes.

**Table 7:** (A) Analysis Model 1 of mistakes and filler words. (B) Analysis Model 2 of mistakes and filler words.

A. Analysis Model 1 of mistakes and filler words.						
*Predictors*	*Estimates*	*SE*	*β*	*95% CI* *[LL, UL]*	*z*	*p*
Percentage of Mistakes						
(Intercept)	−2.17	0.13	−1.80	[0.10, 0.39]	−16.23	<.001
Newcomer’s Arrival^a^	0.24	0.07	−0.12	[0.42, 0.65]	3.33	<.001
**Random Effects**						
Individual Participant Variance	0.27					
Marginal R^2^ / Conditional R^2^	0.032/ 0.609					
Percentage of Filler Words						
(Intercept)	−2.03	0.13	−2.41	[-2.28, -1.77]	−15.32	<.001
Newcomer’s Arrival^a^	−0.26	0.08	−0.13	[-0.42, -0.10]	−3.24	<.010
**Random Effects**						
Individual Participant Variance	0.13					
Marginal R^2^ / Conditional R^2^	0.044/ 0.385					
**B. Analysis Model 2 of mistakes and filler words**						
Change in Percentage of Mistakes						
(Intercept)	1.49	2.39	0.00	[-3.31, 6.30]	0.62	.536
Newcomer’s Percentage of Mistakes	0.23	0.24	0.10	[-0.25, 0.72]	0.97	.339
**Random Effects**						
WebEx Session Variance	0.00					
Marginal R^2^	0.010					
Change in Percentage of Filler Words						
(Intercept)	−0.67	1.40	0.00	[-3.50, 2.16]	−0.48	.636
Newcomer’s Percentage of Filler Words	−0.03	0.17	−0.02	[-0.37, 0.31]	−0.17	.866
**Random Effects**						
WebEx Session Variance	0.00					
Marginal R^2^	0.000					

*SE* = standard error, *β* = standardized coefficient, CI = confidence interval, *LL* = lower limit, *UL* = upper limit*, z* = z-score *p* = p-value.

^a^1 = before newcomer’s arrival, 2 = after the newcomer’s arrival.

The percentage of filler words was significantly and negatively predicted by the newcomer’s arrival (see Table 7A). Participants used significantly fewer filler words after the newcomer’s arrival (before: *M* = 9.04, *SD* = 3.15; after: *M* = 8.15, *SD* = 4.27). Again, however, the newcomer’s percentage of filler words did not significantly predict the change among participants before and after the newcomer’s arrival (see Table 7B). These findings do not support the disruption expectation: Instead, after the newcomer’s arrival, participants used fewer filler words—perhaps a sign of increased speech fluency—regardless of the newcomer’s use of filler words.

#### Clout and authenticity.

To gain a more comprehensive understanding of how the newcomer’s arrival influenced participants, we tested the changes in participants’ speech clout and authenticity. We performed two LMMs each for both clout and authenticity, as they are composite scores ranging from 1 to 99 that fit the normal distribution assumption. The analyses were conducted in R using the *lme4* package [75]. In the first analysis model, the fixed effect and random effects were consistent with previous analyses, examining changes before and after the newcomer’s arrival and accounting for variability in individual participants. The second analysis model further tested whether the newcomer’s speech clout and authenticity predicted changes in participants’ clout and authenticity, respectively. This analysis model also accounted for variability across WebEx sessions.

Speech clout was significantly predicted by the newcomer’s arrival (see Table 8A). Participants had significantly higher speech clout after the newcomer’s arrival (before: *M* = 21.71, *SD* = 18.92; after: *M* = 30.75, *SD* = 25.97). Again, however, the change in participants’ clout was not predicted by the newcomer’s own speech clout (see Table 8B). These results indicated that participants may increase their speech clout to signal authority and differentiate themselves from the group in response to the newcomer’s disruption in social interaction. Importantly, this change was not contingent upon the newcomer’s speech clout.

**Table 8:** (A) Analysis Model 1 of speech clout and authenticity. (B) Analysis Model 2 of speech clout and authenticity.

A. Analysis Model 1 of speech clout and authenticity						
*Predictors*	*Estimates*	*SE*	*β*	*95% CI* *[LL, UL]*	*t*	*p*
Speech Clout						
(Intercept)	12.75	4.61	0.00	[3.64, 21.86]	2.77	.006
Newcomer’s Arrival^a^	8.99	2.83	0.19	[0.07, 0.32]	3.18	.002
**Random Effects**						
Individual Participant Variance	116.19					
Marginal R^2^/ Conditional R^2^	0.038/ 0.254					
Speech Authenticity						
(Intercept)	83.41	5.07	0.00	[73.48, 93.45]	16.44	<.001
Newcomer’s Arrival^a^	−13.01	3.13	−0.26	[-0.38, -0.13]	−4.16	<.001
**Random Effects**						
Individual Participant Variance	121.22					
Marginal R^2^/ Conditional R^2^	0.065/ 0.250					
**B. Analysis Model 2 of speech clout and authenticity**						
Change in Speech Clout						
(Intercept)	10.48	5.98	−0.02	[-0.25, 0.20]	1.75	.086
Newcomer’s Speech Clout	−0.07	0.16	−0.05	[-0.27, 0.18]	−0.44	.660
**Random Effects**						
WebEx Session Variance	156.36					
Marginal R^2^/ Conditional R^2^	0.002/ 0.195					
Change in Speech Authenticity						
(Intercept)	−27.27	10.63	0.01	[-0.21, 0.23]	−2.57	.014
Newcomer’s Speech Authenticity	0.21	0.15	0.16	[-0.08, 0.39]	1.46	.152
**Random Effects**						
WebEx Session Variance	181.35					
Marginal R^2^/ Conditional R^2^	0.027/ 0.214					

*SE* = standard error, *β* = standardized coefficient, CI = confidence interval, *LL* = lower limit, *UL* = upper limit*, t* = t-value *p* = p-value.

^a^1 = before newcomer’s arrival, 2 = after the newcomer’s arrival.

Speech authenticity was significantly predicted by the newcomer’s arrival (see Table 8A). Participants’ speech authenticity significantly decreased after the newcomer’s arrival (before: *M* = 70.42, *SD* = 20.87; after: *M* = 57.36, *SD* = 28.02). As with the other linguistic behaviors, the change in participants’ authenticity was not significantly predicted by the newcomer’s patterns of speech authenticity (see Table 8B). Our findings are consistent with many of the other patterns described above: The newcomer appears to be a disruptive presence, reducing participants’ speech authenticity (perhaps due to participants’ increased self-consciousness and speech regulation).

#### Post-hoc power analysis.

As the study is exploratory in nature, no a priori power analysis was conducted. Thus, a post-hoc power analysis was performed for the groupwise analysis of LSM analysis model 1 (i.e., exploring changes in participants’ LSM following the newcomer’s arrival). Power analysis was not conducted for other linguistic markers or models, as they followed similar structures and were considered sufficiently represented by LSM analysis model 1. The post-hoc power analysis was conducted using the *simr* package in R [76]. Results from the power analysis revealed a very low power estimate (0.00%, CI: 0.00% to 0.37%), indicating that the sample size and small effect size made it challenging to detect meaningful effects. Despite this, participants’ LSM was found to be statistically significantly decreased after the newcomer’s arrival, suggesting a meaningful difference due to the newcomer’s effect. Given the low observed power, it is important to interpret these findings with caution, particularly in the context of naturalistic linguistic data, which is inherently variable across individuals.

### Study 1 discussion

We examined how participants’ linguistic patterns change pre- and post-newcomer’s arrival, focusing on linguistic style matching, speech disfluency (mistakes and filler words), speech clout, and authenticity. Our results indicate that nonstandard-accented participants generally diverged from their own linguistic patterns following the arrival of a standard-accented newcomer. Participants exhibited lower LSM with other participants after the newcomer arrived, and participants who converged more to the newcomer showed greater decreases in their LSM with other participants. Most tellingly, participants’ LSM with the newcomer was significantly and negatively predictive of their LSM with other participants after the newcomer’s arrival.

These patterns suggest a particular trajectory of accommodation processes. Before the newcomer arrived, participants likely became more similar to each other in linguistic styles. After the newcomer’s arrival, however, participants converged toward the newcomer and simultaneously diverged from other participants. These findings make the alternative interpretation—that participants were already diverging before the newcomer arrived—unlikely. If participants had been diverging before newcomer’s arrival, we would expect them to systematically converge toward the newcomer and simultaneously increase similarity with other participants after the newcomer joined. Yet, this pattern is not supported by our results.

Overall, the newcomer’s arrival appeared disruptive, leading participants to diverge from other participants in their linguistic styles while converging more with the newcomer. This hypothesized disruption was further supported by changes in speech patterns after the newcomer’s arrival: increased mistakes, higher speech clout, and lower speech authenticity. Interestingly, we also saw that participants used fewer filler words—showing a language change in a different direction. Importantly, however, these changes in participants’ use of mistakes, filler words, speech clout, and authenticity were not influenced by the newcomer’s own linguistic patterns. Together, these findings suggest that the standard-accented newcomer disrupted group dynamics upon their arrival, rather than merely prompting nonstandard-accented participants to converge toward a new group member.

Past studies have shown that heightened anxiety and feelings of disturbance can increase speech disfluencies [e.g., 58], and the increase in mistakes post-newcomer’s arrival may reflect participants’ mental disturbance and perceptions of disruption. Additionally, increased clout, often associated with confidence, authority, and social dominance [59,60], suggests that participants may have felt the need to assert status or control in response to the newcomer. Meanwhile, the decrease in authenticity suggests heightened self-monitoring and reduced disclosure [65], reinforcing the idea that participants perceived the interaction as disrupted.

A notable exception to the general disruptive pattern was the decrease in filler words. While disturbance has been shown to lead to more disfluencies [e.g., 58], the reduction in filler words may indicate another form of stress. Feeling disrupted, participants may have engaged in greater self-monitoring—evidenced by higher clout and lower authenticity—leading to a more formal speech style. This shift could have similarly reduced the use of filler words, which are typically associated with spontaneous, unfiltered speech [72,77,78]. However, given that the current work cannot speak to this possibility, future research should explore whether this reduction in filler words usage reflects greater self-consciousness or is instead a facilitative effect (as we had initially framed it). To provide a more comprehensive view of how nonstandard-accented participants experience the newcomer’s arrival, Study 2 turns to affective changes that emerge from these interactions.

## Study 2: Changes in positive affect

In Study 2, we explore how a standard-accented newcomer’s arrival impacts nonstandard-accented participants’ perceived interaction quality through their positive affect. Specifically, we examine the dynamics in positive and negative affect among participants.

### Positive and negative affect

We investigate how a standard-accented newcomer’s arrival influences nonstandard-accented participants’ positive affect and perceived interaction quality. Past research found that nonstandard-accented individuals may favor standard accents [e.g., 79,80]. This tendency may lead nonstandard-accented participants to seek social approval. In these scenarios, participants may perceive the standard-accented newcomer’s arrival as facilitative and have stronger positive than negative affect that leads to better perceived interaction quality.

Alternatively, nonstandard accents may serve as group membership cues, potentially fostering positive perceptions during interactions through viewing other nonstandard-accented speakers as ingroup members (see social identity theory [81,82]; see self-categorization theory [83]). Given the negative experiences that nonstandard-accented speakers often encounter [e.g., 10,15,84], they may perceive the standard-accented newcomer’s arrival as disruptive and distance themselves to protect their identity. Consequently, when a standard-accented newcomer enters the social interaction, nonstandard-accented participants may have stronger negative than positive affect, which impacts their perceived interaction quality. Accordingly, we wanted to test two competing expectations: If the newcomer’s arrival facilitates the interaction, positive affect will have a stronger influence than negative affect on post-newcomer perceived interaction quality, controlling for pre-newcomer perceived interaction quality. In contrast, if the newcomer’s arrival disrupts the interaction, negative affect will have a stronger influence than positive affect.

### Study materials

#### Perceived social interaction quality.

After the end of the group interaction, participants were asked to think back and provide separate responses based on the “the first half” and “second half” of the discussion. This allowed us to probe their impressions of the interactions before (“first half”) and after (“second half”) the newcomer arrived. However, because we did not want to call attention to the confederate newcomer, we intentionally did not reference the newcomer’s arrival in framing these questions.

Participants rated the perceived social interaction quality before and after the standard-accented newcomer’s arrival using 11 items. These items include the quality of discussion (e.g., “to what extent do you enjoy the discussion?”), the amount of disclosure (e.g., “how much did you disclose to your discussion mates?”), the amount of engagement (e.g., “to what extent do you think you influenced the discussion?”), and how intimate the discussion is (e.g., “to what extent do you think the discussion was intimate?”) [49]. Participants rated on a 7-point Likert scale, ranging from 1 (*not at all*) to 7 (*very much*). The Cronbach’s α was .87 for both pre- and post-newcomer’s arrival phases. We averaged the items within each participant to obtain a perceived social interaction quality score for before and after the newcomer’s arrival, respectively.

#### Positive and negative affect.

During the post-interaction surveys, participants also rated their positive and negative affect toward the social interaction. Unlike the social interaction quality questions, participants answered these questions about the entire interaction, rather than the first versus second halves of the conversation. These questions were asked on the 20-item Positive and Negative Affect Schedule scale (PANAS [50]). For positive and negative affect, 10 items were asked respectively. Participants responded on a 5-point Likert scale, from 1 (*very slightly or not at all*) to 5 (*extremely*). Examples of positive affects (Cronbach’s α = .87) included “to what extent did you feel interested during the discussion?” and “to what extent did you feel inspired during the discussion?” Examples of negative affects (Cronbach’s α = .77) included “to what extent did you feel distressed during the discussion?” and “to what extent did you feel nervous during the discussion?” The positive and negative affect scores were summed separately within each participant for the entire interaction.

### Results

**Fig 3:**
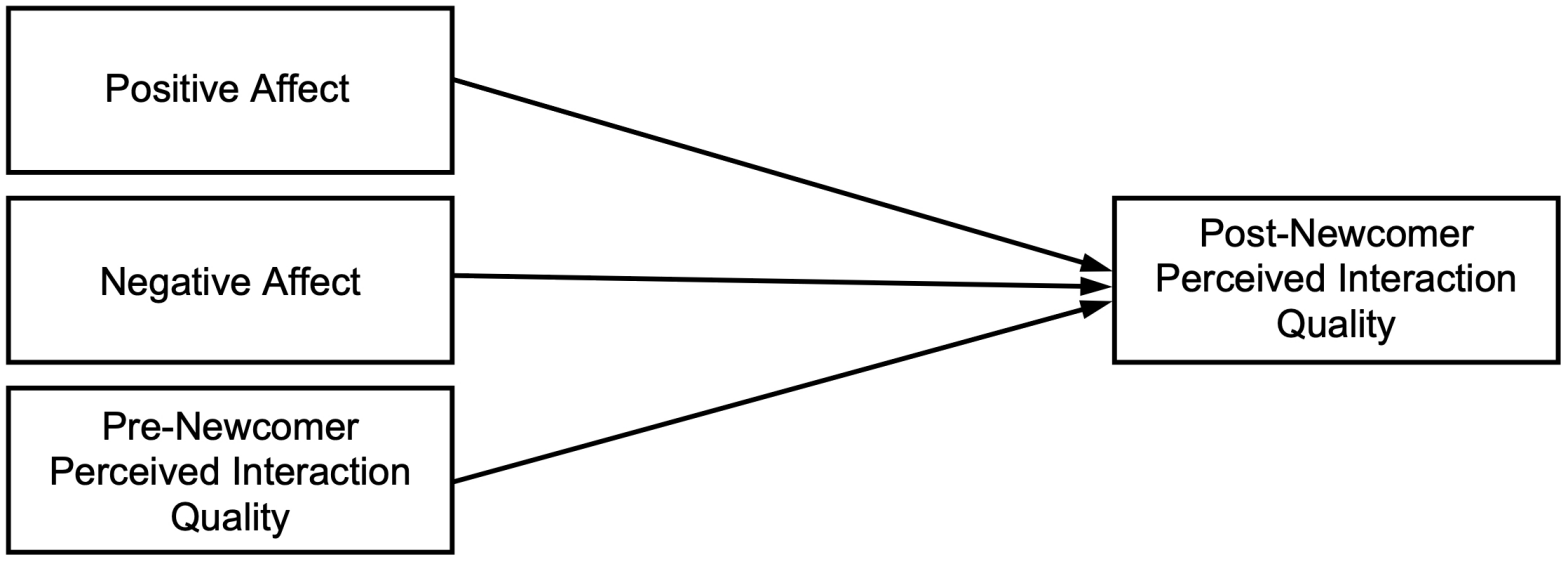
Conceptual model of positive affect.

To test whether positive and negative affect differentially predicted post-newcomer perceived interaction quality based on whether the newcomer facilitated or disrupted the interaction, we performed linear mixed model analysis in R using the *lme4* package [75]. To account for the influences of pre-newcomer perceived interaction quality as a baseline measure, we included it as a predictor, along with positive and negative affect. We also included WebEx session as a random effect. The dependent variable was the post-newcomer perceived interaction quality. See Fig 3 for an illustration of the conceptual model.

Our results showed that positive affect, negative affect, and pre-newcomer perceived interaction quality significantly predicted post-newcomer perceived interaction quality. Together, they explained 56.9% of the variance (*R*^2^ = .569). The individual predictors indicated that positive affect, negative affect, and pre-newcomer perceived interaction quality were all significant predictors. See Table 9 for a summary of the results.

**Table 9:** Positive affect predicts post-newcomer interaction quality.

*Predictors*	*Estimates*	*SE*	*β*	*95% CI* *[LL, UL]*	*t*	*p*
(Intercept)	2.58	0.37	0.00	[1.85, 3.31]	7.06	<0.001
Positive Affect	0.06	0.01	0.51	[0.04, 0.08]	5.89	<0.001
Negative Affect	−0.05	0.02	−0.24	[-0.08, -0.02]	−3.61	<0.001
Pre-Newcomer Perceived Interaction Quality	0.25	0.08	0.27	[0.09, 0.41]	3.12	0.002
**Random Effects**						
σ^2^	0.34					
WebEx Session Variance	0.00					
Marginal R^2^	0.569					

Higher scores in positive affect, negative affect, and pre-newcomer perceived interaction quality indicate greater positivity, greater negativity, and higher perceived interaction quality, respectively. *SE* = standard error, *β* = standardized coefficient, CI = confidence interval, *LL* = lower limit, *UL* = upper limit, *t* = t-value, *p* = p-value.

Comparing the effect of positive and negative affect after controlling for pre-newcomer perceived interaction quality, our results showed positive and negative affect were both significant predictors. Interestingly, positive affect had a stronger effect than negative affect in predicting post-newcomer perceived interaction quality (*β*: | 0.51 | > | −0.24 |). However, both effects showed a consistent pattern: Participants with *more* positive emotion and *less* negative emotion tended to have more positive views of the post-newcomer interaction.

#### Post-hoc power analysis.

Due to the exploratory nature of the study, no a priori power analysis was conducted. However, we performed a post-hoc power analysis for the linear mixed model. The post-hoc power analysis was conducted using *simr* package in R [76]. Results revealed 100% power (CI: 99.63% to 100.0%), indicating that the model had more than sufficient power to detect the observed effect with the present sample size. The high level of power suggests statistical confidence in the robustness of the observed relationship between positive affect and post-newcomer perceived interaction quality.

### Study 2 discussion

Study 2 findings suggest that participants’ positive affect played a stronger role in predicting post-newcomer perceived interaction quality than negative affect, after controlling for pre-newcomer perceived interaction quality. This suggests that participants may have seen the newcomer as facilitating the social interaction, in contrast to the results of many of our linguistic analyses.

According to expectancy violations theory, when expectations about social interactions are violated, individuals experience heightened arousal and engage in an appraisal process. If the violation is perceived positively, it can enhance interaction quality; whereas negative violations can lead to less favorable outcomes [85,86]. When integrated with communication accommodation theory (see [40] for a review), this may suggest that participants’ positive affect is shaped by their expectations about the newcomer’s accent characteristic. Given that all participants shared nonstandard accents, they may have anticipated the newcomer to speak with a nonstandard accent. The fact that the newcomer spoke with a standard accent may have constituted a positive expectancy violation, leading to a stronger positive affect and, in turn, improved perceived interaction quality [87,88]. This positive expectancy violation may suggest the facilitating role of the newcomer.

These findings should be interpreted with some caution, given that our study uses video conference rather than face-to-face communication. Past research has shown differences between these modes of interaction. For example, people can appear less emotionally engaged online [89], and adolescents have been found to report more positive affect and less negative affect when interacting face-to-face compared to online [90]. However, many studies showing systematic differences in online versus face-to-face communication have examined asynchronous communication (e.g., texting, social media). By contrast, studies of more synchronous mediums with greater social presence (e.g., video calls) have shown little or no differences in perceived social connectedness, compared with in-person interaction [91,92]. Future work should continue to examine differences in emotional dynamics and subjective perceptions of outcomes between these settings. Given that our study employed real-time online interactions using a platform familiar to the participants, we expect that our findings should apply to in-person interactions, as well.

## General discussion

Past studies have shown that having a nonstandard accent can lead to negative communication experiences [41,93], as speakers often face challenges such as being mocked, judged, or criticized [14,15,80]. These negative experiences may influence interaction dynamics, particularly when a standard-accented newcomer enters an ongoing interaction among nonstandard-accented speakers. In such interactions, differences in accent status may shape the nature and flow of the interaction. Here, our findings highlight a complex interplay between linguistic patterns and affective experience, offering a richer understanding of how nonstandard-accented participants navigate a standard-accented newcomer’s arrival.

Drawing on communication accommodation theory, the present research introduces a novel, naturalistic paradigm to explore how a standard-accented newcomer facilitates or disrupts ongoing interaction among nonstandard-accented speakers. Moving beyond traditional dyadic and audio-evaluation methods, this work captures real-time linguistic and affective changes within dynamic, small-group discussions. Specifically, Study 1 explores how the linguistic patterns of nonstandard-accented participants change in response to a newcomer’s arrival. Study 2 explores how the newcomer affects nonstandard-accented participants’ perceived interaction quality through positive affect.

Our findings reveal a striking contrast: the standard-accented newcomer’s arrival appeared disruptive based on linguistic patterns but was perceived as facilitative based on participants’ positive affect. This discrepancy may arise from the complex processes underlying linguistic and affective dynamics, which can be impacted by factors as varied as individual motivation [31] to interpersonal closeness [52] to expectations about the situation [85]. Put together, although positive emotions tended to lead to more positive ratings of the interaction, participants dramatically shifted their language when the standard-accented newcomer arrived—becoming more similar to the newcomer’s language style, more formal, and less personal.

A combination of competing forces may help explain this pattern. Divergence is often considered unconscious and driven by discomfort or identity assertion [94]. Upward divergence—that is, moving toward standard-accented speakers and away from nonstandard-accented speakers—can be socially rewarding [93]. Even though interaction quality tended to be marked by more positive emotion, participants appeared to diverge from each other to adapt to the standard-accented newcomer. This could be a strategy to avoid potential discrimination (see [14,15] for discussions on accent-based discrimination). This contrast highlights the complexity of accommodation processes and suggests that perceptions of interaction quality do not always correspond to linguistic behaviors. Future research should further explore how linguistic and affective processes interact to shape accommodation strategies in diverse accent settings.

### Significance and implication

This research pioneers a naturalistic, virtual-interaction paradigm that captures how accent-based social dynamics unfold in real-time—a major departure from traditional perception-focused studies (see [5] for a review). By analyzing subtle shifts in both speech patterns and positive affect as nonstandard-accented strangers navigate a standard-accented newcomer’s arrival, this work reveals how nonstandard-accented speakers actively manage inclusion, identity, and power in everyday interactions.

Our study makes a theoretical contribution by extending the communication accommodation theory to examine how nonstandard-accented speakers accommodate in real-time interactions when a standard-accented newcomer enters the discussion. This approach expands prior research, which has focused on dyadic or stable group interactions [e.g., 51,52], by highlighting how linguistic patterns dynamically shift in response to social context changes. The design—strangers meeting for the first time, with a new person joining midway—captures naturalistic, everyday group dynamics that are often overlooked in laboratory studies. This flexible paradigm can be applied beyond the study of accent to investigate a wide range of real-time social interactions and group dynamics.

Moreover, by examining online social interactions via WebEx, we extend the medium studied within the communication accommodation framework and support newer stages of the theory that emphasize communication beyond face-to-face interactions [40]. Our design simulates modern communication environments, and our findings demonstrate that communication accommodation occurs in online settings. Accents, as social markers of identity and power [e.g., 95,96], influence both linguistic and affective patterns in these interactions.

Since the COVID-19 pandemic, online meetings have become a central mode of communication. By simulating real-time group interaction in a virtual setting, our design captures the interpersonal dynamics typically associated with face-to-face conversation. This offers novel insights into how nonstandard-accented speakers navigate social interactions in digital settings. Although concerns remain that emotional cues may be lost in online interactions, video conference platforms do not consistently reduce perceived social connectedness compared to face-to-face conversations [91,92]. Even though future work should disentangle the differences in accent-diverse communication between face-to-face and digital contexts, the present work’s contributions are especially relevant in today’s increasingly multicultural and multilingual societies, where individuals of different accents regularly interact across both in-person and online platforms. This paradigm also opens new avenues for future research on virtual collaboration, remote work, and hybrid group dynamics.

Additionally, this research introduces linguistic markers as tools to detect micro-level accommodation behaviors in accent-diverse interactions. While prior accent research has focused primarily on single-incident perception tasks (i.e., audio ratings [46,97,98]) or nonstandard-accented speakers’ internalized stigma [e.g., 99], this study captures real-time, interactional responses. Even subtle shifts can reflect the cumulative experience of marginalization among nonstandard-accented speakers—making these momentary changes meaningful, not trivial. Future research could expand this approach by incorporating acoustic voice measures to construct a more comprehensive account of interaction dynamics.

Beyond theoretical implications, our findings hold practical significance. First, newcomer integration is a common challenge in workplace settings, where team members frequently transition in and out [e.g., 1,17]. Given that English serves as a lingua franca in many professional environments, encounters with diverse accents are inevitable [100]. Our study highlights that a standard-accented newcomer’s arrival can disrupt interactions among nonstandard-accented individuals, leading to linguistic divergence among the nonstandard-accented group members. Since communication divergence is often associated with negative consequences [31,101], organizations may benefit from implementing strategies to ease group transitions—particularly when newcomers have different accent backgrounds than existing team members—to foster better collaboration, even when team members hold generally positive feelings toward newcomers.

Second, our findings reveal that when a standard-accented newcomer disrupts an ongoing interaction among nonstandard-accented speakers, these speakers exhibit more linguistic errors, use more clout-related language, and show reduced authenticity in speech. This suggests increased self-monitoring and a shift toward greater formality. These insights have direct implications for second-language acquisition and public speaking training for nonstandard-accented speakers. Often, speakers may experience disruption in public speaking and professional communication (e.g., during a business negotiation). Preparing individuals for such dynamics, such as anticipating increased linguistic errors and shifts in speech style, may help improve overall speech delivery. Training programs could incorporate exercises that simulate these targeted disruptions, allowing speakers to develop strategies for maintaining fluency and confidence in high-stakes communication settings.

### Limitation and future direction

This study is an exploratory investigation of how nonstandard-accented participants accommodate their communication in response to the arrival of a standard-accented newcomer. We adopted a novel approach by integrating both linguistic analyses and self-reported responses. However, due to the exploratory nature of the study, several limitations warrant further investigation.

First, our analysis relied on comparisons before and after the newcomer’s arrival. It is possible that the passage of time, rather than the newcomer’s presence, influenced accommodation. After the newcomer joined, participants may have needed time to establish a new group dynamic, which may drive linguistic accommodation. To mitigate this, we analyzed linguistic patterns for the whole interaction pre-newcomer’s arrival (i.e., the first 10 minutes) and the whole interaction post-newcomer’s arrival (i.e., the last 10 minutes) to capture the new established dynamics.

Importantly, because our study aimed to preserve ecological validity by adopting a naturalistic design, we did not explicitly assess participants’ perceptions of the newcomer’s accent (e.g., whether it was standard or nonstandard). As such, one alternative explanation is that the effects we observed may have stemmed from the mere introduction of a newcomer, independent of their accent. In S1 File Supporting Information, we provide evidence (from a separate study) that nonstandard-accented observers consistently perceived the newcomers—but *not* necessarily the nonstandard-accented participants—as being culturally aligned with the U.S.; this provides converging evidence that this research design successfully made the newcomer’s standard accent a salient aspect of their identity for the nonstandard-accented participants. Nonetheless, future research should explicitly measure participants’ perceptions of the newcomer’s accent to disentangle the effects of group membership change from those of accent-based dynamics. Future research could also address these potential confounds by incorporating additional coding mechanisms, such as qualitative analyses by human observers to assess linguistic patterns and positive affect pre- and post-newcomer’s arrival. In addition, third-party ratings of the newcomer’s accent characteristics would help verify whether participants perceived the accent as standard or nonstandard. By integrating multiple analytic methods, future studies can more rigorously isolate the effects of the newcomer’s accent from those of time passage and the mere presence of a newcomer, thereby enhancing the robustness and interpretability of the findings.

Second, due to misinterpretation of the coding scheme, we discarded Coder 1’s coding and relied solely on the work of Coder 2. This may have reduced the reliability of linguistic pattern results. To address this, the first author reviewed Coder 2’s work, and all coauthors engaged in discussions to ensure the coding remained consistent with the study’s objectives. The final dataset reflects a consensus reached by all parties, mitigating potential bias and maximizing validity. Future studies could enhance reliability by employing additional linguistic analysis tools such as Praat [102] and cross-validating automated analyses with human coding.

Third, real-world interaction dynamics are often far more complex than even the more naturalistic design that we introduced here. People belong to many different social groups. In any given interaction, individuals may share some (e.g., accent, age) but not all (e.g., gender, ethnicity) identities with their conversational partners. Future research could explore the intersection of accent and other identity dimensions to better understand how various social markers influence interaction dynamics.

Relatedly, the present study included participants who self-identified as nonstandard-accented speakers in the U.S., as their subjective perceptions of speaking with a nonstandard accent were central to our aims. However, nonstandard accent is a very broad category that intentionally encompasses substantial variation, and past work shows considerable differences in perception of different nonstandard accents [e.g., 97]. The grammar and vocabulary usage of nonnative speakers may also differ from those of nonstandard-accented native speakers. As this study serves as an initial step to extend the communication accommodation theory to accent-diverse group interactions, we adopted an inclusive approach. Future research could disentangle these differences by focusing on specific accent backgrounds using current study design.

Moreover, the newcomer’s characteristics may have influenced participants’ accommodation strategies. Although we observed that all newcomers exhibited high linguistic style matching, the way they talk and their identity (e.g., gender, race) may have influenced participants’ perceptions. For instance, Kane and Rink [103] found that groups respond more positively to newcomers who use an integrating language-based strategy (i.e., plural pronouns; e.g., we, our) that emphasizes new group identity than to newcomers who use a differentiating language-based strategy (i.e., singular pronouns; e.g., I, my, your, mine) that emphasizes personal identity. Accordingly, the possible moderating effects of the newcomer’s characteristics offer interesting avenues for future research.

Lastly, our findings should be interpreted with caution. Given our naturalistic approach, the study is necessarily observational rather than causal. Specifically, Study 1’s post-hoc power analysis revealed a very low power estimate for detecting the effects of linguistic accommodation, suggesting the study may have been underpowered. Despite this, the observed general disruptive pattern across linguistic markers suggests a meaningful impact of the newcomer’s arrival on linguistic accommodation. In contrast, Study 2 had high power, providing strong evidence for the validity of the findings. The contrasting power analyses highlight the difference between naturalistic linguistic data and self-reported data, especially in real-life settings, which may further explain the varying accommodation patterns found in the present work. Future experimental research could benefit from including a control condition in which no newcomer joins the interaction with a larger sample. This would allow for a more precise assessment of how accommodation changes due to the newcomer’s arrival and could improve the power of the current design.

Despite these limitations, our study is one of the first to examine communication accommodation in real-time online interactions across individuals with different accents. Our findings lay the groundwork for future empirical research on how nonstandard-accented speakers adapt their communication when interacting with standard-accented individuals, offering new directions for studying communication accommodation in diverse accent settings.

## Conclusion

This study explores how nonstandard-accented speakers’ language and emotions change in response to the arrival of a standard-accented newcomer in social interactions. Our findings revealed a nuanced dynamic: Linguistic divergence within nonstandard-accented group members does not necessarily correspond to negative affect and lower perceived interaction quality. Instead, the newcomer’s arrival may function as a social focal point, redistributing attention as the group makeup changes. This shift appears to facilitate perceptions of smoother interactions; however, this improved perception may come at a psychological cost. The observed linguistic shifts may reflect the pressure nonstandard-accented speakers feel to adapt in order to maintain conversational smoothness. These findings highlight the complexity of interaction dynamics in diverse accent settings.

## Supporting information

S1 File Supporting information. Additional analyses on participants and newcomer, and details of coding scheme.
(DOCX)

S1 Data Dataset. Complete dataset for Study 1 and Study 2.
(CSV)
