# Supplementary material for: When nonstandard meets standard: Language and affective dynamics in accent-diverse group interactions
Source: PLoS One. 2026 Jan 28;21(1):e0340873. doi: 10.1371/journal.pone.0340873 (PMC12851482; doi:10.1371/journal.pone.0340873)
Supplement: S1 File — Additional analyses on participants and newcomer, and details of coding scheme. (DOCX) [file pone.0340873.s001.docx]

## When Nonstandard Meets Standard:

## Language and Affective Dynamics in Accent-Diverse Group Interactions

## *Supporting Information*

In the supporting information, additional information regarding the paper’s research methods, analytic procedures, and results is provided. Please see each section for details.

**Mock Participants and Recruitment Adjustments**

Our goal was to recruit at least three participants per WebEx session to examine how individuals interact in social interactions. However, due to specific eligibility criteria and participant availability constraints, we anticipated difficulty in recruitment. As a result, two undergraduate students were invited to repeatedly participate in the WebEx sessions as mock participants to earn research credits. The mock participants were informed that they would engage in several WebEx sessions to discuss their university life with other students. This strategy was implemented to ensure adequate group sizes, as other participants were only asked to join once.

Specifically, mock participants were told that their task was to participate in multiple WebEx discussions. Since they were joining several sessions, they were instructed to simply engage naturally in the discussions without completing additional tasks, unlike other participants. They were also informed that in each session, a late participant might join, whom they may or may not have met before. This was explained as part of the experimental manipulation, aiming to replicate the natural flow of discussions, as students often run late. However, mock participants were not informed about the focus on accent.

The two mock participants were native Chinese speakers who self-identified as speaking with a nonstandard accent in the U.S. They were given the same instructions as other participants and were unaware of the study’s purpose. In most of the WebEx sessions, one mock participant joined the discussion, while two sessions had no mock participant, and three sessions had two mock participants (see Table S1 for detailed session breakdowns). At least one real participant was included in each session, though some sessions had only one real participant. Mock participants did not complete the follow-up survey, as their role was solely to engage in the discussions. To ensure data validity, the transcripts of mock participants were excluded in the analysis.

***Sensitivity Analyses Including Mock Participants***

To examine whether mock participants influenced group dynamics, we ran all Study 1 models with mock participants included (*n* = 50). Results were consistent with the main findings: effect directions and significance levels remained consistent across all models, with two exceptions. First, the pairwise comparison in LSM analysis model 1 became significant (*p* < .001). Second, speech mistake analysis model 1 became insignificant (*p* = .27). These minor variations suggest that although mock participants may have had some localized effects, the overall patterns observed across speech measures were robust.

**Newcomer Backgrounds**

In the present work, the standard-accented newcomers were research assistants who were native speakers of English from the state of Connecticut in the U.S. A total of seven newcomers participated in the WebEx discussion sessions. All newcomers indicated that they did not know any of the participants in the discussion.

Newcomers were instructed to join the discussion 10 minutes after it began (i.e., halfway through the discussion) and were assigned an anonymous ID similar to those of the participants’. The anonymous ID was consistent across all sessions. Upon joining, they were instructed to say: “Hi all, sorry I am late! Do you know where I can tell the experimenter I am here?” After receiving feedback from the participants, newcomers would type “I just arrived” in the WebEx chat. They would then naturally join the discussion with the other participants. Each newcomer participated in 1 to 18 discussions, depending on their availability. Detailed demographics and session information for the newcomers can be found in Table S2.

**Study 1: Coding Scheme for Speech Disfluencies**

In our coding, mistakes indicate that the speech was unclear to the built-in AI transcription service. In other words, rather than directly coding participants’ mistakes, we operationalized speech disfluencies as the discrepancies between the participants’ speech and the AI transcription. This approach was particularly relevant because nonstandard-accented speech often posed greater challenges for AI transcription systems, which tend to be more accurate with standard accents (e.g., [1]). Additionally, participants may have adjusted their speech in response to the newcomer’s entry, either by converging or diverging in their linguistic patterns. These communication adjustments could introduce varying levels of complexities for the AI transcription system, potentially leading to fewer or more transcription errors. By coding discrepancies between the transcripts and actual speech, we capture how communication accommodation affects transcription accuracy, providing nuanced insights into whether participants converged or diverged from each other in their linguistic patterns.

For filler words, we only included filled pauses instead of discourse markers. Filled pauses are short utterances commonly used in spontaneous speech, such as um, uh, er, ah [2, 3]. Discourse markers (e.g., you know), on the other hand, are short phrases that do not contain any grammatical information but are prevalent in natural speech [3, 4]. They serve as transitions between different sections of conversation rather than indicating disfluency [2]. Thus, in the filler words category, we only included filled pauses.

**Study 1: Intercoder Reliability**

The number of mistakes and filler words was coded by two native English-speaking research assistants who were blind to the research design and goals (hereafter *coders*). The coders were given a detailed codebook that explained their procedures. Upon comparing the coding results from Coder 1 and Coder 2, we identified significant discrepancies in Coder 1’s interpretation of the coding scheme. This resulted in low agreement between the two coders (agreement overall was 41.78%; agreement for the mistakes category was 14.86%; agreement for the filler words category was 68.69%). Given these misunderstandings, we opted to rely on Coder 2’s coding for further analysis to ensure greater consistency. To ensure reliability, each coded transcript was subsequently reviewed by the first author. Any disagreements between Coder 2’s coding and the first author’s review were discussed and resolved with all coauthors. In general, Coder 2 and the first author were in strong agreement, with only minor discrepancies. On average, there were 3 to 15 words of disagreement per WebEx session transcript. To put this into context, Coder 2 coded an average of 159.34 words per session (i.e., mistakes, filler words), with a standard deviation of 103.13 (ranging from 10 to 681 words). This iterative process significantly improved the consistency and accuracy of the coding. Although the initial plan to rely on two coders was not feasible due to Coder 1’s misinterpretation, the comprehensive review process resolved this issue, ensuring that the final coding represents a reliable and consistent interpretation of the transcripts.

**Study 1: Eliminate the Newcomer as a Confound**

To investigate the potential confounding effects of individual differences among standard-accented newcomers (*N* = 7), we performed post-hoc LSM analyses on their transcripts using LIWC [5]. We performed a pairwise LSM for each possible pair of newcomers (i.e., two newcomers), and all pairs had LSM scores above 0.80—ranging from 0 (no similarity) to 1 (perfect matching). The average LSM score across all pairs was 0.92, indicating a high level of linguistic alignment across all pairs of newcomers. Because all standard-accented newcomers showed high similarity in language style, we found it unlikely that individual differences in newcomer language would drive the results of our target analyses.

**Saliency of Newcomer’s Standard Accent to Newcomer Perception**

The present study aimed to maximize ecological validity by adopting a naturalistic design. Thus, we did not directly assess participants’ perceptions of the newcomer’s accent (e.g., whether they recognized it as standard or nonstandard). As a result, one alternative explanation is that our findings may have stemmed from the presence of a newcomer, independent of their accent.

To provide converging but indirect evidence that the newcomer’s accent was perceptually salient, we drew on data from a separate study that was conducted in our labs with a comparable population of self-identified nonstandard-accented individuals (hereafter, “observers”). The inclusion criteria were similar to Studies 1 and 2 of the current paper. In this supporting study, 64 observers (*M*_age_ = 21.25, *SD*_age_ = 4.87; 71.9% women, 25.0% men, 1.6% non-binary) were instructed to watch short snippets of interaction recordings deriving from the present study. Observers were not told anything about the interactants’ linguistic backgrounds; that is, they did not know the interactions involved individuals from different accent backgrounds. For each interactant, observers listed the culture(s) they believed the person was affiliated with in an open-ended format. The observers had lived in the U.S. for an average of 6.10 years (*SD* = 6.24).

Across all recording snippets, there were 396 total possible ratings (302 for nonstandard-accented interactants and 96 for standard-accented newcomers). The response rate was high: participants generated 289 (95.7%) ratings for nonstandard-accented interactants and 88 (91.7%) ratings for standard-accented newcomers. Observers labeled the standard-accented newcomer as having an “American” cultural affiliation in 93.2% (*n* = 82) of their ratings, compared to 32.9% (*n* = 95) of ratings of “American” cultural affiliation for nonstandard-accented interactants. Although cultural affiliation is not a direct measure of accent perception, this pronounced difference (93.2% vs. 32.9%) suggests that observers—who shared similar accent backgrounds with the participants of Studies 1 and 2—perceived the standard-accented newcomers as culturally distinct from nonstandard-accented interactants.

Together, these results provide indirect support that the newcomer’s accent was likely highly salient among participants during their interaction. As such, the newcomer’s mere presence is likely not solely responsible for the effects observed in Studies 1 and 2, although it may have been one of the factors involved in shaping the rich and naturalistic interactions in the present work. Nonetheless, we recognize that definitive evidence requires direct measurement of accent perceptions in future work, which we discuss in the manuscript’s Limitation and Future Direction section.

**References**

1. Nacimiento-García E, Díaz-Kaas-Nielsen HS, González-González CS. Gender and accent biases in AI-based tools for Spanish: A comparative study between Alexa and Whisper. Appl Sci. 2024;14(11):4734. <https://doi.org/10.3390/app14114734>
2. Fox Tree JE. Folk notions of um and uh, you know and like. Text Talk. 2007;27(3):297–314. <https://doi.org/10.1515/TEXT.2007.012>
3. Laserna C, Seih YT, Pennebaker JW. Um . . . Who like says you know. J Lang Soc Psychol. 2014;33(3):328–38. <http://dx.doi.org/10.1177/0261927X14526993>
4. Fox Tree JE, Schrock JC. Basic meanings of you know and I mean. J Pragmat. 2002;34(6):727–47. <https://doi.org/10.1016/S0378-2166(02)00027-9>
5. Ireland ME, Pennebaker JW. Language style matching in writing: Synchrony in essays, correspondence, and poetry. J Pers Soc Psychol. 2010;99(3):549–71. <https://doi.org/10.1037/a0020386>

**Tables**

Table S1

Breakdowns of WebEx Session Participants

| Session | Newcomer | Mock Participant | Real Participant | Session | Newcomer | Mock Participant | Real Participant |
| --- | --- | --- | --- | --- | --- | --- | --- |
| 001 | Newcomer 2 | 1 | 1 | 029 | Newcomer 2 | 1 | 2 |
| 003 | Newcomer 6 | 1 | 2 | 030 | Newcomer 6 | 1 | 2 |
| 004 | Newcomer 1 | 1 | 2 | 031 | Newcomer 2 | 1 | 2 |
| 005 | Newcomer 6 | 1 | 2 | 032 | Newcomer 1 | 1 | 2 |
| 007 | Newcomer 1 | 1 | 2 | 033 | Newcomer 2 | 1 | 3 |
| 008 | Newcomer 2 | 1 | 3 | 035 | Newcomer 2 | 1 | 2 |
| 009 | Newcomer 5 | 1 | 2 | 036 | Newcomer 2 | 1 | 2 |
| 010 | Newcomer 1 | 1 | 2 | 037 | Newcomer 6 | 2 | 1 |
| 011 | Newcomer 6 | 1 | 2 | 038 | Newcomer 2 | 1 | 3 |
| 012 | Newcomer 6 | 1 | 2 | 039 | Newcomer 2 | 1 | 2 |
| 013 | Newcomer 1 | 1 | 1 | 040 | Newcomer 6 | 1 | 2 |
| 014 | Newcomer 1 | 1 | 2 | 041 | Newcomer 2 | 1 | 2 |
| 015 | Newcomer 2 | 1 | 1 | 042 | Newcomer 2 | 1 | 2 |
| 016 | Newcomer 6 | 1 | 2 | 043 | Newcomer 4 | 1 | 2 |
| 017 | Newcomer 2 | 0 | 2 | 044 | Newcomer 3 | 1 | 2 |
| 018 | Newcomer 2 | 1 | 2 | 045 | Newcomer 7 | 1 | 3 |
| 019 | Newcomer 1 | 1 | 3 | 046 | Newcomer 3 | 1 | 3 |
| 020 | Newcomer 6 | 2 | 1 | 048 | Newcomer 2 | 1 | 2 |
| 021 | Newcomer 6 | 1 | 3 | 049 | Newcomer 4 | 1 | 2 |
| 022 | Newcomer 6 | 1 | 2 | 050 | Newcomer 6 | 1 | 2 |
| 023 | Newcomer 2 | 1 | 2 | 051 | Newcomer 3 | 1 | 2 |
| 024 | Newcomer 1 | 0 | 4 | 052 | Newcomer 3 | 1 | 3 |
| 025 | Newcomer 6 | 1 | 2 | 053 | Newcomer 2 | 1 | 1 |
| 027 | Newcomer 2 | 1 | 2 | 054 | Newcomer 6 | 1 | 1 |
| 028 | Newcomer 1 | 1 | 3 |  |  |  |  |

*Note.* The columns for mock and real participants indicate the number of these participants.

Table S2

Newcomer Demographics and Session Information

| Newcomer | Participated Sessions | Gender | Place of Origin | Native Language | Education |
| --- | --- | --- | --- | --- | --- |
| Newcomer 1 | 9 | Man | Connecticut, USA | English | University Junior |
| Newcomer 2 | 18 | Woman | Connecticut, USA | English | University Senior |
| Newcomer 3 | 4 | Man | Connecticut, USA | English | University Sophomore |
| Newcomer 4 | 2 | Woman | Connecticut, USA | English | University Junior |
| Newcomer 5 | 1 | Woman | Connecticut, USA | English | University Graduate |
| Newcomer 6 | 14 | Woman | Connecticut, USA | English | University Junior |
| Newcomer 7 | 1 | Woman | Connecticut, USA | English | University Sophomore |

*Note.* There were 49 WebEx discussion sessions in total. All newcomers were given the same instructions and used “PP021” as their anonymous participant ID. The “Participated sessions” column indicate the number of sessions the newcomer engaged in.
